# A novel role for kynurenine 3-monooxygenase in mitochondrial dynamics

**DOI:** 10.1371/journal.pgen.1009129

**Published:** 2020-11-10

**Authors:** Daniel C. Maddison, Mónica Alfonso-Núñez, Aisha M. Swaih, Carlo Breda, Susanna Campesan, Natalie Allcock, Anna Straatman-Iwanowska, Charalambos P. Kyriacou, Flaviano Giorgini

**Affiliations:** 1 Department of Genetics and Genome Biology, University of Leicester, Leicester, LE1 7RH, United Kingdom; 2 Leicester School of Allied Health Sciences, Faculty of Health and Life Sciences, De Montfort University, Leicester, LE1 9BH, United Kingdom; 3 Core Biotechnology Services, Adrian Building, University of Leicester, University Road, Leicester, LE1 7RH, Leicestershire, United Kingdom; University of Washington, UNITED STATES

## Abstract

The enzyme kynurenine 3-monooxygenase (KMO) operates at a critical branch-point in the kynurenine pathway (KP), the major route of tryptophan metabolism. As the KP has been implicated in the pathogenesis of several human diseases, KMO and other enzymes that control metabolic flux through the pathway are potential therapeutic targets for these disorders. While KMO is localized to the outer mitochondrial membrane in eukaryotic organisms, no mitochondrial role for KMO has been described. In this study, KMO deficient *Drosophila melanogaster* were investigated for mitochondrial phenotypes *in vitro* and *in vivo*. We find that a loss of function allele or RNAi knockdown of the *Drosophila* KMO ortholog (*cinnabar*) causes a range of morphological and functional alterations to mitochondria, which are independent of changes to levels of KP metabolites. Notably, *cinnabar* genetically interacts with the Parkinson’s disease associated genes *Pink1* and *parkin*, as well as the mitochondrial fission gene *Drp1*, implicating KMO in mitochondrial dynamics and mitophagy, mechanisms which govern the maintenance of a healthy mitochondrial network. Overexpression of human KMO in mammalian cells finds that KMO plays a role in the post-translational regulation of DRP1. These findings reveal a novel mitochondrial role for KMO, independent from its enzymatic role in the kynurenine pathway.

## Author summary

Mitochondria are subject to quality control mechanisms to maintain homeostasis, regularly undergoing fission and fusion to rescue damaged organelles or segregate regions that are damaged beyond repair which are then cleared by mitophagy. These quality control mechanisms have been implicated in a number of neurodegenerative diseases, including familial Parkinson’s disease, which can be caused by mutations in two mitophagy governing genes, PINK1 and PRKN. The kynurenine pathway is a pathway through which dietary tryptophan is metabolised. The pathway has multiple branchpoints and an imbalance in flux through these branches has been associated with neurodegenerative disease. The enzyme kynurenine 3-monooxygenase (KMO) sits at a critical branchpoint controlling balance in the pathway. KMO is localised to mitochondria, yet to date any mitochondria-specific function is unknown. In this study, we demonstrate that KMO plays a role in mitochondrial fission via the fission factor DRP1 and that KMO deficiency leads to changes in mitochondrial morphology and function. Manipulation of KMO in *Drosophila melanogaster* also demonstrates an interaction with PINK1 and PRKN. Intriguingly, these interactions appear to be independent of the known enzymatic role of KMO in the kynurenine pathway, demonstrating a novel and independent function of the protein.

## Introduction

**Fig 1:**
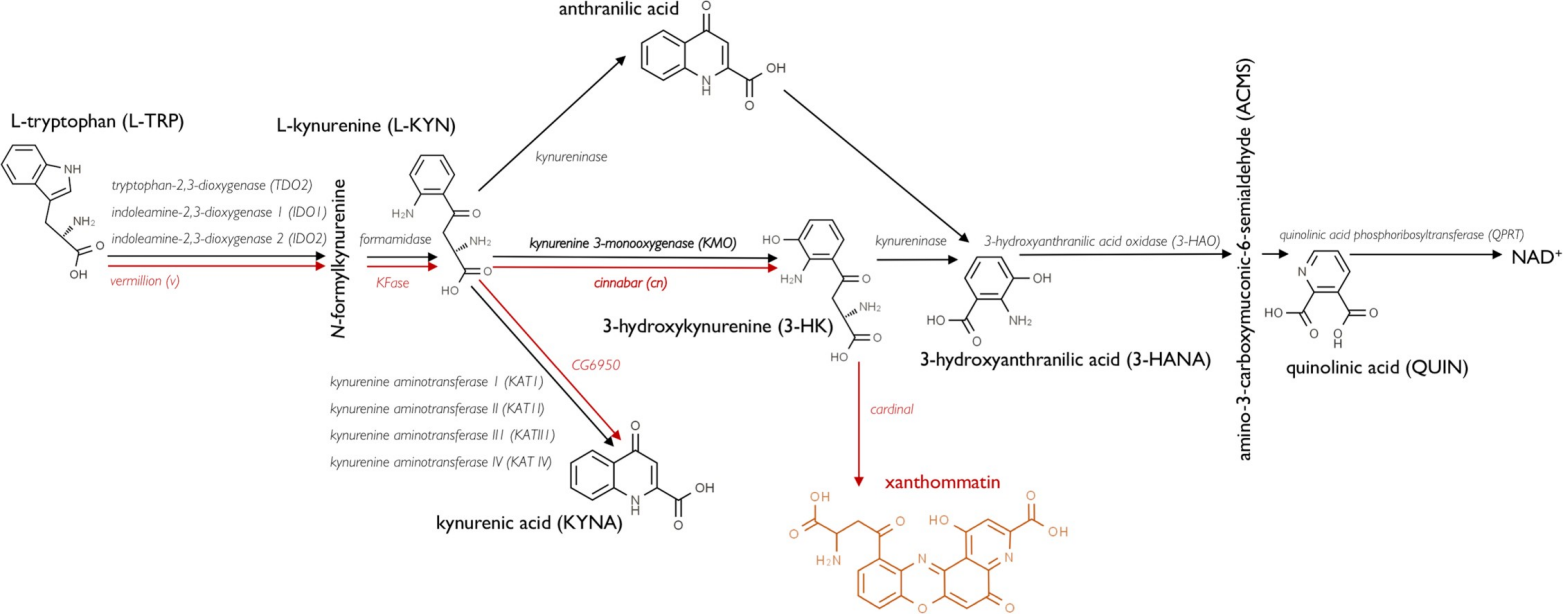
The kynurenine pathway (KP) in mammals and *Drosophila*. The KP is the major route of tryptophan (TRP) degradation in mammals, with >95% of the essential amino acid degraded through the pathway. TRP is metabolised by IDO1/2 or TDO2 to produce L-kynurenine (L-KYN). L-KYN is metabolised through two distinct branches of the pathway; by kynurenine aminotransferases (KATs) to produce kynurenine acid (KYNA) or by KMO to produce 3-hydroxykynurenine (3-HK), 3-hydroxyanthranillic acid (3-HANA), quinolinic acid (QUIN) and ultimately nicotinamide adenosine dinucleotide (NAD+). Genes encoding enzymes in *Drosophila* are in red text. Flies lack homologues for 3-hydroxyanthranilic acid (3-HAO) and quinolinic acid phosphoribosyltransferase (QPRT), so the pathway is uncoupled from NAD^+^ synthesis in flies. 3-HK is converted into the brown ommochrome pigment xanthommatin by phenoxazinone synthetase (cardinal) in *Drosophila*.

The kynurenine pathway (KP) (Fig 1) is the major route of tryptophan metabolism in eukaryotes and has been implicated in the pathology of several human diseases, particularly brain disorders including neurodegeneration, schizophrenia and depression [1,2]. Although the connection between the KP and pathology varies between disorders, one common feature is the imbalance in metabolites produced through distinct branches of the KP. For this reason, enzymes that control the rate of flux through the pathway have become candidate drug targets for the treatment of these diseases. Indeed, both genetic and pharmacological manipulation of KP enzymes is protective in yeast, *Drosophila melanogaster* and mammalian models of neurodegeneration [3–6]. The enzyme kynurenine 3-monooxygenase (KMO) synthesizes the metabolite 3-hydroxykynurenine (3-HK), thereby regulating a key step in the KP which governs the relative balance between several neuroactive metabolites (Fig 1). Several drugs inhibiting KMO have been developed which hold promise as potential therapeutic agents for neurodegenerative disease [3,6,7].

In eukaryotic cells KMO is a mitochondrial protein, localising to the outer mitochondrial membrane (OMM) due to a hydrophobic C-terminal domain [8]. Although the catalytic properties of KMO and its role in disease have been extensively studied, any biological relevance of its mitochondrial localisation are not understood. In a *Drosophila* S2R+ cell genome-wide RNAi screen, the *KMO* homologue *cinnabar (cn)* was identified as a modulator of mitochondrial morphology and the recruitment of the familial Parkinsonism related protein Parkin (PRKN) to depolarised mitochondria [9]. Mitochondrial dysfunction is typically accompanied by dissipation of membrane potential, which causes PTEN-induced kinase 1 (PINK1) to accumulate on the OMM. PINK1 phosphorylates Ser65 of ubiquitin molecules at the OMM, which promotes recruitment and tethering of PRKN [10,11]. PINK1 also phosphorylates Ser65 of the ubiquitin-like domain of PRKN, activating its E3 ubiquitin ligase activity, leading to the extension of polyubiquitin chains on the OMM. Ubiquitin and PRKN are further phosphorylated by PINK1, creating a positive-feedback loop of mitochondrial PRKN recruitment, activation of its E3 ubiquitin ligase activity and decoration of mitochondria with polyubiquitin chains [12–15]. PRKN ubiquitinates a number of targets on the OMM, including the mitofusins MFN1 and MFN2 (encoded by *Marf* in *Drosophila*), targeting them for proteasomal degradation [16,17].

MFNs are important factors in the regulation of mitochondrial dynamics, an umbrella term for the mechanisms which control fission and fusion of mitochondria. While MFNs are responsible for fusion of the OMM [18–22], the mitochondrial dynamin-like GTPase OPA1 is responsible for fusion of the inner mitochondrial membrane [23]. Dynamin related protein (DRP1), a GTPase which forms ring structured polymers, causes mitochondrial fission by constriction of the organelles [24,25]. Mitochondrial dynamics and mitophagy have been well characterised in *Drosophila*. Indeed, *parkin* and *Pink1* mutant flies exhibit a range of mitochondrial and morphological phenotypes, including elongated and aggregated mitochondrial networks, a decrease in respiratory capacity, ATP synthesis and locomotor ability, as well as dopaminergic neuron and muscle degeneration [26–29]. An increase in gene dosage of *Drp1* or a reduction in *Marf* or *Opa1* rescues *Pink1* and *parkin* mutant *Drosophila* phenotypes [30–32], revealing that the mechanisms governing mitochondrial dynamics and mitophagy are intrinsically linked. PINK1 regulates DRP1 GTPase activity in human cells by phosphorylating OMM-bound A-kinase anchoring protein 1 (AKAP1), releasing its interaction with protein kinase A (PKA) and resulting in a decrease in DRP1 phosphorylated at the Ser637 residue, and thus an increase in mitochondrial fission [33]. This is thought to facilitate the compartmentalisation and selective mitophagy of damaged regions of the mitochondrial network, as opposed to wholesale elimination [34].

Here we report a range of phenotypes related to mitophagy, mitochondrial dynamics and energy metabolism in *cinnabar* deficient *Drosophila*, including elongated mitochondrial morphology, an increase in total mitochondrial mass and a decrease in oxidative phosphorylation. These alterations appear to be independent of KMO enzymatic activity, as they are not rescued by 3-HK supplementation. Genetic epistasis experiments reveal an interaction between *cinnabar*, *Pink1* and *parkin*: *cn* loss of function (LOF) induces partial developmental lethality in both *Pink1* and *parkin* mutant *Drosophila*. Furthermore, overexpression of either *cinnabar* or human *KMO* is sufficient to drastically rescue climbing defects in *Pink1* but not *parkin* mutant flies. Decreased locomotor ability in *cn* flies is reversed upon overexpression of *Drp1*, as has been previously observed in *Pink1* and *parkin* mutants. Finally, we show that overexpression of KMO in HEK 293T cells modulates phosphorylation of DRP1, resulting in increased mitochondrial fission, offering an insight into the mechanisms by which KMO modulates mitochondrial form and function.

## Materials and methods

### Cell culture

*Drosophila* Schneider’s S2 cells (Invitrogen, UK) were cultured in Hyclone SFX-Insect Medium (GE Healthcare, UK) supplemented with 10% (v/v) fetal bovine serum (FBS) (Gibco, UK), 100 U/mL penicillin and 100 μg/mL streptomycin. Cells were incubated at 25 ^o^C and passaged every 3–4 days, when they reached a confluence of ~1 x 10^7^ cells/mL. Human embryonic kidney (HEK 293T) cells were routinely cultured in GlutaMAX Dulbecco’s Modified Eagle Medium (Gibco) supplemented with 10% (v/v) FBS (Gibco), 100 U/mL penicillin and 100 μg/mL streptomycin. Cells were incubated at 37 ^o^C, 5% CO_2_ and passaged every 3–4 days, when they reached a confluence of ~80%. Cells were detached by incubation for 3–5 min at 37 ^o^C in 5 mM EDTA- phosphate-buffered saline (PBS).

### Drosophila stocks, husbandry and compound supplementation

Canton S (BS64349), *cn*^*3*^_,_ *v*^*36f*^ (BS142), *Act5CGAL4/TM6B* (BS3954), *Act5CGAL4/CyO* (BS25374) and *FLAG-FlAsH-HA-Drp1*. *Ki*^*1*^*(BS42208*) stocks were obtained from the Bloomington *Drosophila* Stock Center, Indiana University, USA. The *cn* RNAi line (#105854) was obtained from the KK library of the Vienna *Drosophila* Resource Center. *Pink1*^*B9*^ [28] and *park*^*25*^ [27] lines were kind gifts from Alexander Whitworth (MRC Mitochondrial Biology Unit, University of Cambridge, UK). The RNAi control line used contains the VDRC pKC26 cloning vector inserted at the VIE-260B landing site (Breda et al., 2016). The UAS-control line expresses FLPG5 under control of 5XUAS, to control for GAL4 sequestration in the UAS overexpression lines.

All experimental flies were maintained at 25 ^o^C on maize-based medium (yellow cornmeal (72 g/l), glucose (79.3 g/l), brewer's Yeast (50 g/l), agar (8.5 g/l), propionic acid (0.3% v/v), 20% Nipagen in EtOH (1.35% v/v)), under a 12:12 light:dark regime. For feeding experiments, 3-HK (Sigma) and kynurenic acid (KYNA) (Sigma) were dissolved in ddH_2_O. The KMO inhibitor Ro 61–8048 (Sigma) was dissolved in dimethyl sulfoxide (DMSO) (0.001% final). Each compound was added to ~50 ^o^C medium at the desired concentration and mixed thoroughly before dispensing into vials.

### Generation of cn and hKMO overexpression lines

The *cinnabar* coding sequence was amplified from S2 cell cDNA and hKMO was amplified from the pcDNA3.1-hKMO expression vector [35]. Primers were designed to incorporate XhoI or NotI (5’) and XbaI (3’) restriction sites for cloning into the pUASt-attB vector [36]. PCR products were separated by size by agarose-gel electrophoresis and purified using the MinElute Gel Extraction Kit (Qiagen), according to manufacturer’s instructions. Purified products were blunt-end ligated into the pJET1.2 subcloning vector using the CloneJET PCR Cloning Kit (Thermo Scientific, UK) before cloning into the pUASt-attB vector. *cn* and *hKMO* containing pUASt-attB plasmids were injected into the *y*^*1*^.w* M{vasint.*Dm}ZH2A; attP40* line by the Fly Facility, Department of Genetics, University of Cambridge.

### 3-HK level measurements

Tissue from 10 fly heads was subjected to sonication in ACN: UP solution, followed by centrifugation to generate supernatants which were used for analysis. 3-HK levels were assessed by HPLC with tandem mass spectrometry (MS/MS) detection performed by Charles River Discovery Groningen as previously described (6).

### Rapid iterative negative geotaxis (RING) assay

Flies were placed 10 per cohort in 18.4 x 2.3 cm transparent plastic cylinders, with a threshold marked 8 cm vertically. Flies were startled by firm tapping on a rubber mat. 10 s later, the number of flies above the 8 cm threshold was counted. The procedure was repeated 10 times per cohort of flies, with 60 s recovery time between iterations. Experiments were performed in a 25 ^o^C controlled room between ZT3-ZT5.

### Lifespan assay

0–24 hrs post-eclosion, male flies were placed 10 per cohort into plastic vials. Flies were counted and transferred to fresh medium (without anaesthetisation) every 2–3 days.

### Eclosion and defective thorax scoring

To quantify developmental lethality, F1 progeny of each genotype were counted twice-daily for 10 days, the maximum window before the potential eclosion of F2 progeny. 12–24 hrs post-eclosion, male *Pink1*^*B9*^ or *park*^*25*^ flies were anaesthetised and thoraces were assessed using a dissection microscope. The assay was binary, in the sense that flies were scored as either possessing the phenotype or not.

### High-resolution respirometry

Single flies were homogenised in 80 μl of MiR05 respiration medium (EGTA (0.5 mM), MgCl_2_^.^6 H_2_O (3 mM), K-lactobionate (60 mM), Taurine (20 mM), KH_2_PO_4_ (10 mM), HEPES (20 mM), sucrose (110 mM), BSA (1 g/l)). The homogenate from each fly was added to 1920 μl of MiR05 medium in a single chamber of the Oroboros 2k Oxygraph. Complex I-coupled respiration was initiated by the addition of substrates pyruvate (final concentration 2 mM) and glutamate (5 mM) with ADP (1–5 mM). Complex I + II-linked respiration was activated through the addition of succinate (10 mM). ETS capacity was measured by uncoupling of oxygen reduction from ATP synthase activity via the addition of ~6 0.1 μM titrations of the protonophore carbonyl cyanide m-chlorophenyl hydrazine (CCCP). Complex II ETS was measured after the addition of the Complex I inhibitor rotenone (0.5 μM). The Complex IV inhibitor sodium azide (100 mM) was finally added to measure extracellular (non-mitochondrial) oxygen consumption. All values were subject to background subtraction of this value.

### Citrate synthase activity assay

Five whole flies were homogenised for 60 s in 100 μl CellLytic MT Cell Lysis Reagent (Sigma) and debris was cleared from the lysate by centrifugation at 10,000x g for 10 mins at 4 ^o^C. The lysate was diluted 5-fold in lysis reagent and used for both citrate synthase and BCA protein content assays.

Citrate synthase (CS) activity was assayed using the Citrate Synthase Assay Kit (Sigma) on a 96 well plate according to manufacturer instructions, with each sample measured in triplicate. CS is an enzyme which catalyses a key reaction in the TCA cycle, where citrate is produced from acetyl CoA and oxaloacetate. A by-product of this reaction is coenzyme A (CoA-SH), which reacts with 5,5'-dithiobis-(2-nitrobenzoic acid) (DTNB) in this assay to produce 2-nitro-5-benzoic acid (TNB). The concentration of TNB in each sample was measured by light absorbance at 412 nm. A FLUOstar plate reader (Omega) was used to monitor the change in absorption of 412 nm wavelength light after addition of oxaloacetate to each sample and the rate of change in absorbance during the linear phase of the reaction was used to calculate CS activity (μM / ml / min). CS activity of each sample was normalised to total protein content, quantified by BCA assay (Thermo Scientific, UK).

### Transmission electron microscopy (TEM)

The heads from newly eclosed flies were dissected in PBS at room temperature (RT) and proboscises were removed with sharp forceps to aid penetration of fixative. Heads were fixed in 2% paraformaldehyde (Sigma, UK), 2.5% glutaraldehyde (Agar Scientific, UK), 0.1 M sodium cacodylate buffer (pH 7.4) and subsequently fixed in 1% osmium tetroxide (Agar Scientific, UK) / 1.5% potassium ferricyanide (Sigma, UK). Fixed heads were washed three times in de-ionised H_2_O, followed by dehydration steps in ethanol (Sigma, UK) (30%, 50%, 70%, 90% and 100%). Heads were taken through a graded series of propylene oxide: Spurr’s low viscosity resin (Agar Scientific, UK), then embedded in 100% resin, which was polymerized at 60 ^o^C for 16 hrs. Ultra-thin (~70 nm) sections of the eye were cut using a Ultracut E Ultramicrotome (Leica Microsystem, Milton Keynes, UK), collected onto copper mesh grids and stained first with 2% aqueous uranyl acetate for 30 mins, then lead citrate for 2 min. Sections were viewed on a JEM-1400 TEM (JEOL Ltd, Welwyn Garden City, UK) at an accelerating voltage of 100 kV and images of 10,000x magnification were captured using a Megaview III digital camera with iTEM software (EMSIS, Germany).

Mitochondria were traced manually in FIJI [37] and measured using the *“Measure”* function, which calculated aspect ratio (major axis/minor axis), circularity (4π x (area/perimeter^2^) and Feret’s diameter (the greatest distance between two points) of each organelle. Form factor was calculated as 1/circularity, so that a perfect circle gives a form factor = 1, and the less circular an object is, the higher its form factor.

### RNA isolation and cDNA synthesis

RNA was isolated using TRIzol reagent (Ambion, UK), according to the manufacturer’s instructions. Briefly, samples (10 whole flies or ~1x10^6 S2 cells) were homogenized/lysed in 1 mL TRIzol. 200 μL chloroform was added and samples were shaken vigorously before centrifugation at 13,000 g for 15 mins at 4 ^o^C. The aqueous phase was carefully separated and thoroughly combined with equal volumes of isopropanol. Precipated RNA was pelleted by centrifugation at 13,000 g for 10 mins at 4 ^o^C. Pellets were twice washed in 70% ethanol and allowed to dry before resuspension in nuclease-free H_2_O. Concentration and quality of RNA was assessed by spectrophotometry using the Nanodrop 8000 (Thermo Scientific, UK). Removal of any remaining genomic DNA from the RNA was achieved using the TURBO DNAse kit (Ambion, UK). cDNA was synthesized using the QuantiTect Reverse Transcription kit (QIAGEN, UK) according to the manufacturer’s protocols, by random priming.

### dsRNAi synthesis

A *cn* dsRNA template was obtained from the Sheffield RNAi Screening Facility, UK. Other dsRNAi template sequences were obtained from the Heidelberg HD2.0 library [38] and amplified from S2 cell cDNA, using the following T7 flanked primers (*firefly luciferase* F: *TAATACGACTCACTATAGGG*CCTGGTTCCTGGAACAATTGC, R: *TAATACGACTCACTATA*GGGCGGAGTTCATGATCAGTGC; *parkin* F: *TAATACGACTCACTATAGGG*TATTCAGACGCTCCTCGCTT, R: *TAATACGACTCACTATAGGG*TTTTGTACGCAAAATGCTGG; *cn* F: *TAATACGACTCACTATAGGG*GAGGGTATGCAGAGCTCCAG, R: *TAATACGACTCACTATAGGG*TTTGTACGAGTACCGGGAGG) and Phusion High-Fidelity DNA Polymerase (Thermo Scientific, UK). Templates were used to synthesise dsRNA by *in vitro* transcription reactions, using the T7 Megascript kit (Ambion, UK), according to the manufacturer’s instructions. Concentration and quality of RNA was assessed by spectrophotometry using the Nanodrop 8000 (Thermo Scientific, UK).

### Quantitative PCR (qPCR)

qPCR reactions were performed on a LightCycler 480 system (Roche, UK) using Maxima SYBR Green master mix (Thermo Scientific, UK). Total reaction volume was 10 μl, with forward and reverse oligonucleotide concentrations of 330 nM. Oligonucleotide sequences (*cn*—PP1629, *parkin—*PP20972, *rp49—*PD41810) were obtained from DRSC FlyPrimerBank [39]. Four technical replicates were used for each sample and a control reaction in which no reverse transcription was carried out was also included. Crossing points (Cp) were determined by the second derivative method using LightCycler 480 Software (Roche, UK). For relative expression quantification, raw fluorescence data of technical replicates with Cp values within 0.5 cycles of each other were averaged for each sample. The amplification efficiency of each reaction was calculated using the qpcR package in R Studio (Ritz & Spiess, 2008) by fitting sigmoidal curves to the raw fluorescence data, using the *pcrbatch* function. The fold-change ratio of expression was calculated using the *ratiobatch* function. Statistical significance was calculated using a pairwise fixed random reallocation test, similar to that used by REST software (Pfaffl, 2002). Briefly, efficiency values are tied to Cp values and randomly shuffled between experimental and control samples for 1000 permutations. For each permutation, a fold-change expression ratio is calculated and compared to the value generated from the original data. The number of permutations which produce a fold-change greater than, equal to or smaller than the original data is used to produce a *P* value, representing the probability that the fold-change calculated from the original data is due to chance.

### Mitotracker Red FM staining, laser confocal imaging and image processing

For S2 cell RNAi experiments, cells were transfected with dsRNA using Effectene reagent. 60 hrs post-transfection, cells were seeded at 2 x 10^5^/dish in glass-bottomed 35 mm dishes (Ibidi) coated with Concanavalin A. 12 hrs later, cells were stained with Mitotracker Red FM (100 nM in complete Schneider’s medium) for 30 mins. The media was replaced with fresh complete media and cells were imaged live at 25°C on an Olympus FV1000 scanning confocal microscope (60x UPlanSAPO Olympus objective, numerical aperture = 1.2, zoom = 4, Kalman = 6). HEK 293T cells were seeded at 1 x 10^5^ cells per dish in glass-bottomed dishes coated with poly-l-ornithine. 48 hrs post-transfection, cells were washed in PBS and given fresh complete media. 72 hrs post-transfection, cells were stained with Mitotracker Red FM (100nM in complete media) for 30 mins. Medium was replaced with fresh complete DMEM and cells were imaged live at 37 ^o^C, 5% CO_2_ on an Olympus FV1000 scanning confocal microscope (60x UPlanSAPO Olympus objective, numerical aperture = 1.2, zoom = 4, Kalman = 6). Images were deconvolved using Huygen’s Professional, then processed in FIJI using the following macro code:

*run("Z Project*…*"*, *"projection = [Max Intensity]");*
*run("Subtract Background*…*"*, *"rolling = 10");*
*run("Enhance Contrast*…*"*, *"saturated = 0*.*001");*

For quantification of mitochondrial parameters, the following macro was applied:

run("Make Binary");
*run("Analyze Particles*…*"*, *" circularity = 0–0*.*99 show = [Bare Outlines] display exclude summarize");*

Aspect ratio (major axis/minor axis) and circularity (4π x (area/perimeter^2^) values were produced by the *Analyze Particles* function. Form factor was calculated as 1/circularity, so that a perfect circle gives a form factor = 1, and the less circular an object is, the higher its form factor.

### Mitochondrial fractionation

Mitochondria were isolated from cells using the Mitochondrial Isolation Kit for Cultured Cells (Mitosciences), according to manufacturer’s protocol. Cells were pelleted at 200x g for 5 min at RT, media was aspirated and cell pellets were snap frozen in liquid N_2_. Pellets were thawed at 37 ^o^C for 1 min, resuspended in Kit Buffer A and ruptured using a dounce homogeniser (30 strokes). Nuclear fractions were removed by centrifugation, then mitochondrial and cytosolic fractions were separated by a second centrifugation. The mitochondrial pellet was resuspended in RIPA supplemented with Halt Phosphatase Inhibitor Cocktail (Thermo Scientific, UK).

### SDS-PAGE and immunoblotting

Proteins were separated on Novex 10% Tris-glycine gels (Invitrogen) and were transferred to nitrocellulose membrane by wet transfer. Membranes were blocked with 5% (w/v) milk protein (or bovine serum albumin (BSA) for phospho-sensitive assays) in TBS-T (0.1% TWEEN20) 1 hr. Membranes were incubated with antibodies in 5% (w/v) milk or BSA TBS-T (0.1% TWEEN20) at 4 ^o^C for 16–24 hrs (primary antibodies) and 1 hr RT (secondary antibodies), with gentle agitation. Membranes were washed 3 x 10 min in TBS-T (0.1% TWEEN20) after primary and secondary antibody incubations. HRP-conjugated secondary antibodies were detected using SuperSignal West PICO Plus Chemiluminescent substrate (Thermo Scientific, UK) and imaged with the GeneGnome XRC imaging system (Syngene). Membranes were stripped using Restore Plus Stripping Buffer (Thermo Scientific, UK) at 37 ^o^C for 15 minutes, followed by incubation with secondary antibody for 1 hr at RT and Chemiluminescent substrate. Membranes were imaged to assess complete removal of primary antibody before reprobing. The following antibodies were used: rabbit anti-Marf [17] 1:1000, rabbit anti-DRP1 (Cell Signaling, #8570) 1:1000, rabbit anti-DRP1 pSer616 (Cell Signaling, #3455) 1:1000, rabbit anti-DRP1 pSer637 (Cell Signaling, #4867) 1:1000, mouse anti-GAPDH (Santa Cruz Biotechnology, sc32233) 1:200), rabbit anti-KMO (Proteintech, 10698-1-AP) 1:1000 and mouse anti-VDAC1 (Cell Signaling, #4866) 1:1000.

### Statistical analysis

qPCR analyses were performed using R Studio, while all other statistical analyses were performed using Prism 7 (GraphPad). Details of tests performed on individual experiments are described in figure legends.

## Results

### Elongated mitochondria are observed in *cinnabar* deficient fly models

**Fig 2:**
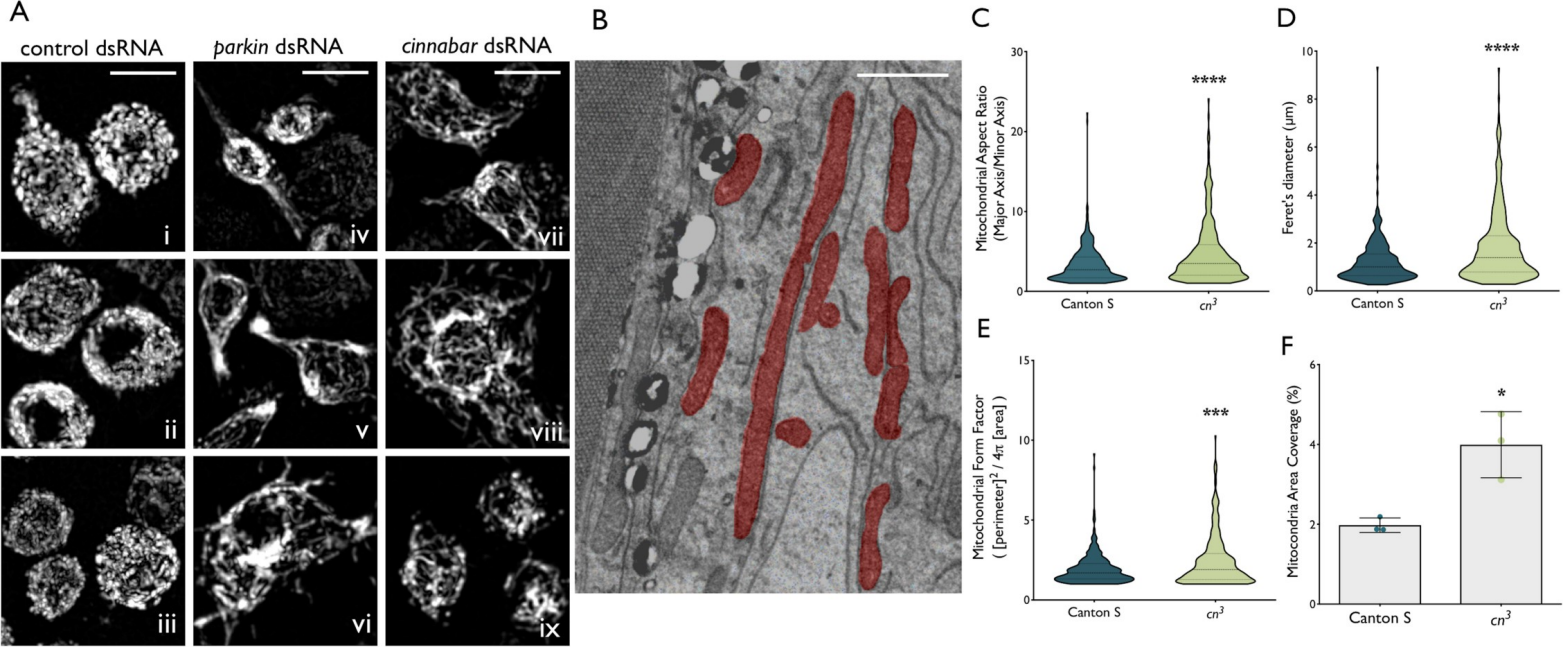
Mitochondria are elongated due to *cinnabar* reduction *in vitro* and *in vivo*. **(A)** Mitochondrial morphology is altered due to *cinnabar* (vii-ix) and *parkin* (iv-vi) dsRNA treatment in *Drosophila* S2 cells, compared to non-targeting control dsRNA cells (i-iii) (scale bars = 5 μm). **(B)** Transmission electron microscopy images from eye tissue of *cn*^*3*^ flies. (scale bar = 1 μm). **(C)** Aspect ratio (major axis length / minor axis length). **(D)** Feret’s diameter (distance between two most distal points). **(E)** Form factor (degree of branching, calculated as the inverse of circularity) (median ± 95% CI, Mann-Whitney U test, *** *P* < 0.001, **** *P* < 0.0001, n = 3, > 350 total mitochondria analyzed per group,). **(F)** Mitochondrial area coverage (percentage of total area analyzed covered by mitochondria. Mean ± SD, Welch’s t test, * *P* < 0.05, n = 3).

We sought to corroborate the observation that dsRNAi-mediated knockdown of *cinnabar* causes a shift in mitochondrial dynamics towards more elongated organelles in *Drosophila* immortalised cells [9]. S2 cells were transfected with dsRNAi constructs targeting firefly *luciferase* (control), *cinnabar* or *parkin* and after 72 hrs were stained with MitoTracker Red and imaged live by confocal microscopy. *cinnabar* silencing (~80% knockdown, S1A Fig) resulted in an elongation of the mitochondrial network compared with cells treated with the control dsRNAi construct (Fig 2A), although this phenotype was not as pronounced as that from *parkin* silencing (~98% knockdown, S1A Fig), which caused both elongation and aggregation of the mitochondrial network.

To investigate whether these effects are present *in vivo*, transmission electron microscopy (TEM) was employed to compare mitochondrial morphology in the *cinnabar* null *cn*^*3*^ line to the Canton S wild-type strain. As *cinnabar* expression is highly enriched in the *Drosophila* compound eye [40], we selected this structure for imaging. Longitudinal sections of the eye were generated to obtain images best reflecting mitochondrial length (Fig 2B). The aspect ratio and Feret’s diameter were increased in *cn*^*3*^ flies compared to Canton S control flies, reflecting mitochondrial elongation arising from KMO deficiency (Fig 2C & 2D); Mann-Whitney test, *P* < 0.0001). Form factor was also increased in *cn*^*3*^ flies (Fig 2E, Mann-Whitney test, *P* = 0.0013), indicative of increased branching of the mitochondrial network. Mitochondria also covered a higher percentage of the total area measured in *cn*^*3*^ flies compared to Canton S (Fig 2F, Welch’s t test, *P* = 0.046), suggesting an increase in mitochondrial mass.

### Mitochondrial respiratory capacity and locomotor activity are decreased in *cn* flies, independent from 3-HK synthesis

**Fig 3:**
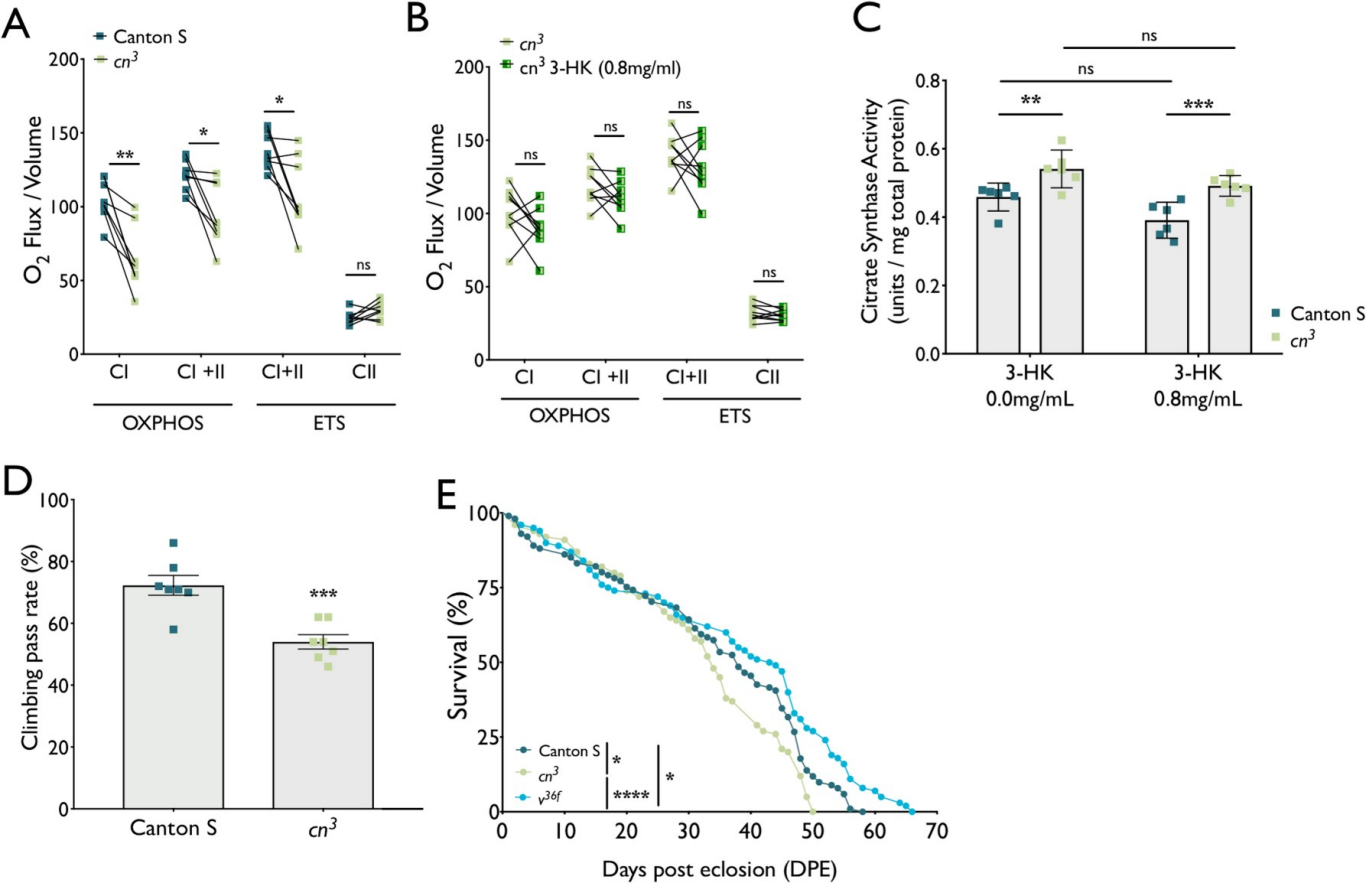
Changes in TCA cycle, OXPHOS, locomotor ability and lifespan in *cn*^*3*^ flies. **(A)** Respiratory capacity is reduced in *cn*^*3*^ mutants flies. (mean ± SEM; paired t test, Holm-Sidak post hoc, ** *P* < 0.01, * *P* < 0.05, n = 8). **(B)** 3-HK supplementation does not rescue *cn*^*3*^ respirometry phenotypes (mean ± SEM; paired t test, Holm-Sidak post hoc, ns = not significant, n = 9) **(C)** Citrate synthase activity is increased *cn*^*3*^ amorphs even when supplemented with 3-HK (mean ± SEM; two-way ANOVA, Tukey post hoc, ** *P* < 0.01, *** *P* < 0.001, ns = not significant, n = 6). **(D)** Climbing ability is reduced in *cn*^*3*^ mutants (mean ± SEM; student t-test, *** *P* < 0.001, n = 6). **(E)** Longevity is recued in *cn*^*3*^ but enhanced in *v*^*36f*^ mutants (n = 100, Mantel-Cox test, * *P* < 0.05, **** *P* < 0.0001).

We next investigated if the observed alterations in mitochondrial morphology and content observed in *cn*^*3*^ flies were correlated with changes in mitochondrial respiration and energy metabolism. High resolution respirometry was employed to assess the performance of the mitochondrial electron transfer system (ETS) and oxidative phosphorylation (OXPHOS). *cn*^*3*^ flies exhibited a significant decrease in the OXPHOS capacity of ETS complex I (CI) and complex I & II combined (CI+II) in comparison with Canton S control flies. CI+II combined ETS capacity was also reduced upon uncoupling of OXPHOS from ATP synthase activity by CCCP treatment (Fig 3A). However, the ETS capacity of CII alone was not significantly different between *cn*^*3*^ and Canton S flies. We validated the above observations using a second *cn* deficient model, in which *cn* was knocked-down ubiquitously by GAL4^Act5C^-driven RNAi. *cn*^*RNAi*^ flies exhibited a ~70% reduction in *cn* mRNA expression versus controls (S1B Fig). *cn*^*RNAi*^ flies exhibited similar defects in CI OXPHOS and CI+II OXPHOS and ETS respiratory capacities as those observed in *cn*^*3*^ flies (S1C Fig). To investigate whether the observed effect could be due to a change in KP metabolite levels caused by absence of *cn*, the product of KMO activity—3-HK—was supplemented in the food of *cn*^*3*^ amorphs sufficient to restore it to physiological levels [4]. Supplementation of 3-HK in the diet of *cn*^*3*^ flies had no significant effect on respiratory capacity (Fig 3B), indicating that the decrease in respiratory capacity of *cn*^*3*^ flies is independent of KMO enzymatic activity.

The citrate synthase (CS) assay was used to infer total mitochondrial mass [33,41,42]. *cn*^*3*^ flies showed a significant increase in CS activity compared to Canton S controls (Fig 3C), supporting the observation by TEM that mitochondrial mass is greater in *cn*^*3*^ flies. 3-HK supplementation did not significantly affect CS activity in either *cn*^*3*^ or Canton S flies (Fig 3C), again implying that KMO enzymatic function is unrelated to its involvement in mitochondrial phenotypes.

To assess the consequences of these mitochondrial phenotypes on behaviour, we used the repetitive iteration negative geotaxis (RING) assay as a metric of locomotor ability. *cn*^*3*^ flies showed a significant decrease in locomotion compared to Canton S at 7 days post eclosion (Fig 3D), which was replicated in *GAL4*^*Act5C*^-driven *cn*^RNAi^ flies at 7–35 days (S1D Fig). *cn*^*3*^ flies also exhibited a significantly shorter lifespan than Canton S controls (Fig 3E). Conversely, flies carrying a mutation in the fly TDO orthologue (*vermillion*, *v*^*36f*^) had a significantly longer lifespan than either *cn*^*3*^ or Canton S flies, indicating that the effect of both mutations on lifespan is likely to be independent of 3-HK synthesis, which is lacking in both mutants.

### *Cinnabar* genetically interacts with *Pink1* and *parkin* in a mechanism independent of KP metabolism

**Fig 4:**
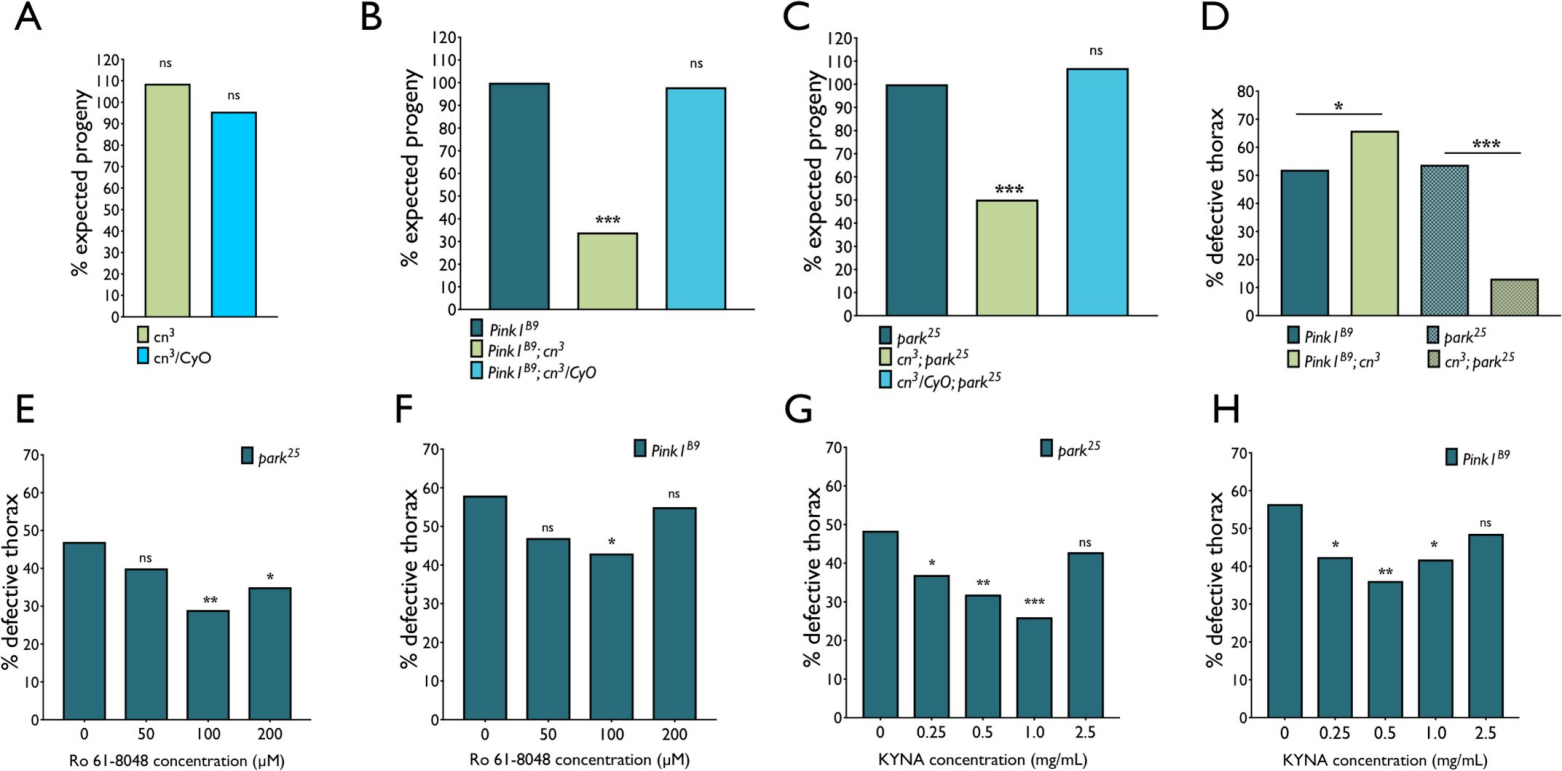
Homozygous *cn* LOF causes synthetic lethality in *Pink1*^*B9*^ and *park*^*25*^ flies. **(A)** *cn*^*3*^*/CyO* in a *w*^*-*^ wild-type background were self-crossed. The expected Mendelian ratio of *cn*^*3*^ to *cn*^*3*^*/CyO* flies is 1:2. *CyO* is homozygous lethal. % expected progeny was calculated from the total number of progeny. **(B)** Crosses were set up to generate *Pink1*^*B9*^ flies carrying the *cn*^*3*^ allele. The ratio of *Pink1*^*B9*^ to *FM6* flies is 1:1. % expected progeny was calculated as the proportion of *Pink1*^*B9*^ male progeny in relation to *FM6* (χ^2^ test, 1 d.f., *** *P* < 0.001). **(C)** Expected progeny was calculated in proportion to the number of corresponding *park*^*25*^*/TM6B* flies that eclosed (χ^2^ test, 1 d.f., *** *P* < 0.001). **(D)** Penetrance male defective thorax phenotype of Day 1 *Pink1*^*B9*^ or *park*^*25*^ males combined with homozygous *cn*^*3*^. Penetrance in the *Pink1*^*B9*^*; cn*^*3*^ and *cn*^*3*^*; park*^*25*^ genotypes was compared to the *park*^*25*^ *stock*. *χ* ^*2*^ *test*, *1 d*.*f*., * P < 0.*0125*, *** P < 0.*00025*. **(E)** Effect of KMO inhibitor Ro 61–8048 on defective thorax phenotype in *park*^*25*^ and *Pink1*^*B9*^ **(F)** flies (*χ* ^*2*^ *test*, *1 d*.*f*., * P < 0.*0167*, *** P < 0.*0003*) **(G)** Effect of KYNA supplementation on defective thorax phenotype in *park*^*25*^ and *Pink1*^*B9*^ **(H)** flies.

Increased mitochondrial length and mass accompanied by a reduction in respiratory capacity and locomotor ability suggest imbalanced mitochondrial dynamics in *cn* flies, associated with a decrease in mitochondrial fission and mitophagy. This implicates KMO in mitochondrial quality control mechanisms which are regulated in part by the familial Parkinson’s disease (PD) associated proteins PINK1 and PRKN [43]. To probe for an interaction between *cn* and *Pink1* or *parkin* in *Drosophila*, epistasis experiments were performed in which the *cn*^*3*^ amorph was introduced into *Pink1* and *parkin* mutant backgrounds and both *cn* and human *KMO (hKMO)* were overexpressed in these mutants. *cn*^*3*^ null mutants were crossed into *Pink1*^*B9*^ and *park*^*25*^ LOF mutant backgrounds, and the genotype of eclosed progeny were counted for both double-mutant and controls (Fig 4A–4C). When *cn*^*3*^*/CyO* flies were crossed to each other, there was no deviation from the expected 0.33 ratio of *cn*^*3*^ homozygotes that eclosed (Fig 4A). Hemizygosity in *Pink1*^*B9*^ or homozygosity in *park*^*25*^ male flies alone was also not sufficient to produce a deviation from the expected genotype ratio (Fig 4B and 4C). However, the proportion of total *Pink1*^*B9*^ or *park*^*25*^ progeny homozygous for *cn*^*3*^ was significantly lower than the expected, indicating partial developmental lethality in *Pink1*^*B9*^*; cn*^*3*^ and *cn*^*3*^*; park*^*25*^ flies (Fig 4B and 4C).

We next investigated if *cn* LOF affected the defective thorax phenotypes found in *Pink1* and *parkin* mutants, caused by degeneration of flight muscle tissue and indentations to the thorax. The phenotype has previously been reported in 50–90% of young *Pink1*^*B9*^ *and park*^*25*^ mutants [26,44,45], but is not present in *cn*^*3*^ mutants. In our study, ~60% of *Pink1*^*B9*^ flies and ~50% of *park*^*25*^ flies exhibited thoracic indentations 12–24 hrs post-eclosion. Strikingly, when combined with *cn*^*3*^ homozygosity, the proportion of *Pink1*^*B9*^ flies with the phenotype increased to ~65%, whereas penetrance was dramatically reduced to ~20% in *park*^*25*^ flies (Fig 4D). Upon supplementation with an inhibitor of KMO enzymatic activity (Ro 61–8048), similar protection was observed in *park*^*25*^ flies at 100 μM and 200 μM concentrations (Fig 4E). In *Pink1*^*B9*^ mutants, significant protection was also conveyed with 100 μM Ro 61–8048, the opposite modulation of the phenotype to that observed in the *cn*^*3*^ amorph. These findings indicate that whilst a decrease in KMO enzymatic activity might explain the decrease in phenotype penetrance in *park*^*25*^ flies, the increase in penetrance in *Pink1*^*B9*^*; cn*^*3*^ flies is independent of KMO enzymatic activity.

To further investigate whether modulation of the phenotype is caused by a shift in KP flux as a result of *cn* LOF, *Pink1*^*B9*^ and *park*^*25*^ mutants were fed KYNA at a range of concentrations, to mimic the increase in flux through the KYNA-producing branch of the KP in *cn*^*3*^ flies and Ro 61–8048 flies [4,5]. Indeed, 0.25, 0.5 and 1.0 mg/mL concentrations of KYNA supplementation caused a significant decrease in penetrance of the defective thorax phenotype in *park*^*25*^ and *Pink1*^*B9*^ mutants (Fig 4G and 4H). No effect was observed at 2.5 mg/mL KYNA supplementation, indicating that at higher levels, the protective effect of KYNA is abolished. The protective effect of KMO enzymatic inhibition thus appears to be conveyed through elevated KYNA, which is sufficient to account for the amelioration observed in *cn*^*3*^*; park*^*25*^ but not the exacerbation observed in *Pink1*^*B9*^*; cn*^*3*^ flies.

**Fig 5:**
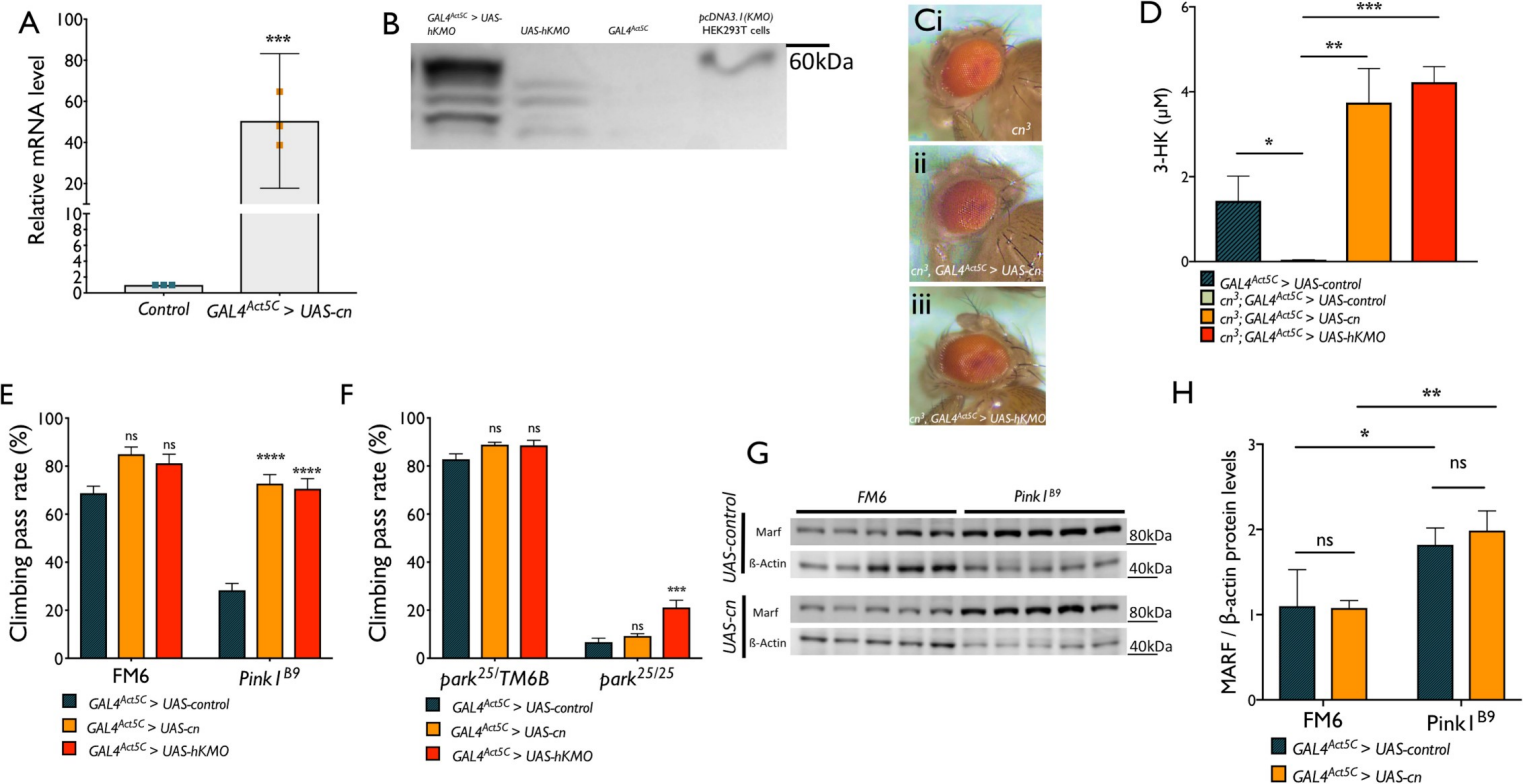
*cn or hKMO overexpression rescues climbing ability in Pink1*^*B9*^ and *park*^*25*^ flies while not affecting Marf levels. **(A)** Overexpression of *UAS-cn* was driven by *GAL4*^*Act5C*^_._ Expression of *cn* was assessed by qPCR of cDNA from whole flies **(**mean ± SD, randomisation test, *** *P* < 0.0001). **(B)** Overexpression of *UAS-hKMO* was driven by *GAL4*^*Act5C*^_._ Expression of hKMO was assessed by SDS-PAGE and immunoblotting of 20 μg whole fly protein extract and ran alongside protein from HEK 293T cells transiently transfected with pcDNA3.1(KMO). **(C)** *GAL4*^*Act5C*^ *–*driven overexpression of *UAS-cn* or *UAS-hKMO* is sufficient to rescue the eye colour phenotype in *cn*^*3*^ mutant flies. **(D)** 3-HK levels in heads from control flies, *cn*^*3*^, or *cn*^*3*^ overexpressing *cn* or *hKMO* constructs (mean ± SD, one way ANOVA, Tukey *post hoc*, * *P* < 0.05, ** *P* < 0.001, *** *P* < 0.0001, n = 5). **(E, F)** *cn or hKMO* expression rescues *Pink1* and *parkin* climbing phenotypes (mean ± SEM; two-way ANOVA, Tukey *post hoc*, *** P < 0.001, **** P < 0.0001, ns = not significant, 10 flies per n, n = 8–10). **(G)** Marf levels are not affected by *cn* overexpression. **(H)** β-actin normalised Marf levels are increased in *Pink1*^*B9*^ flies compared to FM6 (*Pink1*^*+*^) but unchanged by *cn* overexpression (Mean ± SD, two-way ANOVA, Tukey *post hoc*, * *P* < 0.05, ** *P* < 0.01, ns = not significant, n = 5).

To further investigate a potential interaction between KMO and the PINK1/PRKN pathway, we tested *cn* or human *KMO (hKMO)* overexpression in *Pink1* and *parkin* mutants. To achieve this, we cloned *cn* and *hKMO* cDNA sequences into the pUASTattB vector, which was then microinjected into embryos carrying the *attP40* landing site. Recombinant flies were crossed to flies carrying the *GAL4*^*Act5C*^ driver and progeny showed a ~60-fold upregulation in *cn* mRNA assessed by qPCR, or KMO protein assessed by immunoblotting (Fig 5A and 5B). *GAL4*^*Act5C*^ -driven expression of these constructs was sufficient to rescue both 3-HK levels and eye colour in *cn*^*3*^ mutant flies, demonstrating the conversion of kynurenine to 3-HK and therefore the presence of functional KMO protein (Fig 5C and 5D).

Overexpression of these constructs resulted in a striking rescue of locomotor ability in *Pink1*^*B9*^ flies, assessed by the RING assay (Fig 5E). In *park*^*25*^ flies, a more modest but significant rescue was observed with overexpression of *hKMO* (Fig 5F). These results could be interpreted to suggest that KMO operates downstream of PINK1 but upstream or independent of Parkin in the initiation of mitophagy. To investigate this further, we asked whether overexpression of *cn* influenced the levels of Marf, which is increased in *Pink1* and *parkin* mutant flies, due to the lack of Parkin recruitment to mitochondria leading to ubiquitination and proteasomal degradation of Marf [46]. We found β-actin normalised Marf levels to be ~1.9-fold higher in *Pink1*^*B9*^ flies compared to FM6 (*Pink1*^*+*^) progeny from the same cross (Fig 5G and 5H). Upon overexpression of *cn*, no significant differences in Marf levels were observed in either *Pink1*^*+*^ or *Pink1*^*B9*^ flies, indicating that the protection conveyed by *cn* overexpression in *Pink1*^*B9*^ flies is independent of mitochondrial Parkin recruitment and ubiquitination of Marf.

### *Drp1* upregulation reverses climbing phenotype of *cn*-deficient flies

**Fig 6:**
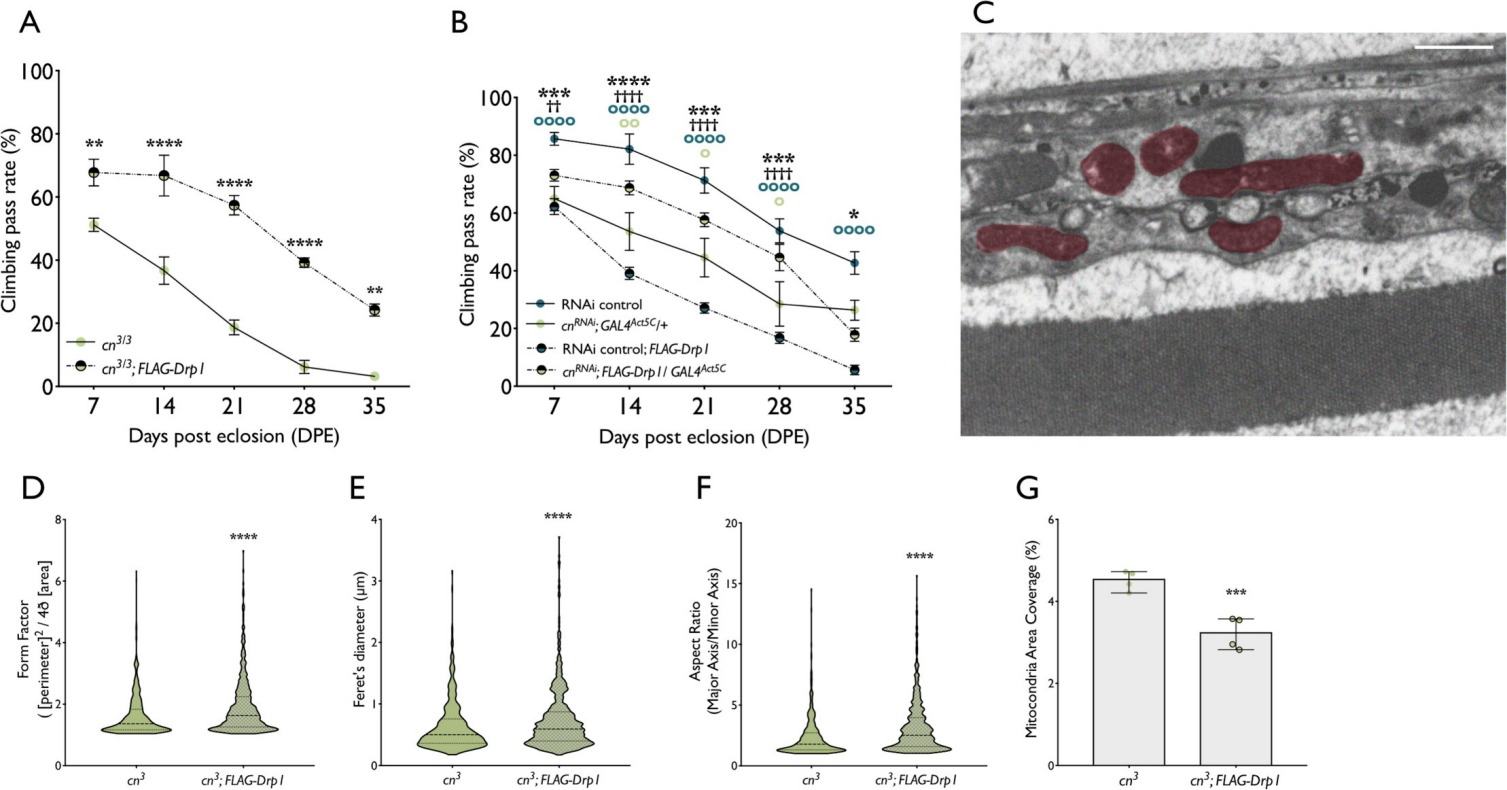
*cn* genetically interacts with *Drp1*. **(A)** Overexpression of *Drp1* via the *FLAG-Drp1* transgene improves climbing ability in *cn*^*3*^ flies. The *FLAG-Drp1* transgene, under control of the endogenous *Drp1* promoter, was introduced into *cn*^*3*^ flies. Climbing ability was assessed in flies aged 7–35 days (mean ± SEM, Two-way ANOVA, Tukey *post hoc*, n = 5–10). **(B)** Overexpression of *Drp1* via the *FLAG-Drp1* transgene improves climbing ability in *cn* RNAi flies (mean ± SEM, Two way ANOVA, Tukey *post hoc*, * *P* <0.05; ** *P* < 0.01; *** *P* <0.001; **** *P* <0.0001. * = *P value* RNAi control vs *cn*^*RNAi*^*; GAL4*^*Act5C*^*/+*. † = *P* value *RNAi control; FLAG-Drp1* vs *cn*^*RNAi*^*; GAL4*^*Act5C*^*/FLAG-Drp1*. **o** = *P* value RNAi control vs RNAi control; *FLAG-Drp1*. **o** = *P* value *cn*^*RNAi*^*; GAL4*^*Act5C*^ vs *cn*^*RNAi*^*; GAL4*^*Act5C*^*/FLAG-Drp1*. n = 8–10 (10 flies per n). **(C)** Longitudinal sections from *cn*^*3*^*; FLAG-Drp1* flies. (**D)** Form factor of traced mitochondria (median ± 95% CI, Mann-Whitney test, **** *P* < 0.0001, n = 4, >500 mitochondria analysed per group). (**E)** Feret’s diameter of traced mitochondria (median ± 95% CI, Mann-Whitney test, **** *P* < 0.0001, n = 4, >500 mitochondria analysed per group). (**F)** Aspect ratio of traced mitochondria (median ± 95% CI, Mann-Whitney test, **** *P* < 0.0001, n = 4, >500 mitochondria analysed per group). **(G)** % area coverage of traced mitochondria (mean ± SD, t test, *** *P* < 0.05, n = 4).

dsRNA silencing of *Drp1* in S2 cells results in elongated mitochondria [47] and heterozygous *Drp1* LOF is lethal in *Pink1* and *parkin* mutant flies [31,48]. The elongated mitochondrial phenotype observed in *cn*-deficient flies and cells, accompanied by the genetic interactions among *cn*, *Pink1* and *parkin*, which appear to be independent of Marf, indicate a potential overlap in function between *cn* and *Drp1*. To investigate a potential functional interaction between KMO and DRP1, *Drp1* was overexpressed in *cn*^*3*^ flies. Overexpression was achieved *via* a genomic construct which provides an extra copy of *Drp1*, and therefore increases its expression by ~50% [49]. This transgenic line was previously used to rescue mitochondrial morphology and muscle degeneration in *Pink1*^*B9*^ flies [32] and causes a milder upregulation of Drp1 than overexpression via a UAS construct [50]. Introduction of the additional copy of *Drp1* significantly and dramatically improved locomotor ability in *cn*^*3*^ flies at all ages assayed (Fig 6A). A similar effect was observed in *cn* RNAi flies but introduction of the allele to the RNAi control had a detrimental effect at all ages assayed, suggesting enhanced fission is detrimental in a wild-type background (Fig 6B).

Given the improved climbing performance of *cn*^*3*^ flies upon introduction of an additional *Drp1* allele, we next investigated whether this amelioration correlated to altered mitochondrial dynamics in *cn*^*3*^ flies. Mitochondrial morphology in longitudinal sections of the retina was assessed by TEM (Fig 6C). A significant difference in mitochondrial morphology was observed between *cn*^*3*^ and *cn*^*3*^*; Drp1* flies, where paradoxically, *Drp1* upregulation caused a significant increase in mitochondrial aspect ratio, Feret’s diameter and form factor (Fig 6D–6F). However, the increased mitochondrial area observed in *cn*^*3*^ flies was reduced upon Drp1 overexpression, indicating that an increase in DRP1 activity may promote mitophagy in animals lacking KMO activity (Fig 6G).

### KMO modulates DRP1 post-translational regulation

Although an additional copy of *Drp1* improved climbing performance in *cn* null and knockdown flies, it had a detrimental effect upon the RNAi control group, and *cn*^*RNAi*^ flies performed better than the control group upon introduction of the transgene. This indicates that loss of *cn* compensates for the effect of raised DRP1 levels. DRP1 activity is regulated by a number of post-translational modifications, including phosphorylation at two serine residues, which correspond to Ser616 and Ser637 in human DRP1 isoform 1. Phosphorylation at the Ser616 residue by Cyclin dependant kinase 1(CDK1)/cyclin B promotes DRP1 GTPase activity and thus mitochondrial fission during mitosis [51]. Conversely, Ser637 phosphorylation by protein kinase A (PKA) inhibits DRP1 GTPase activity and mitochondrial fission [52,53]. Upon mitochondrial depolarization, release of Ca^2+^ into the cytosol activates calcinuerin, which dephosphorylates DRP1 at Ser637 [53,54].

**Fig 7:**
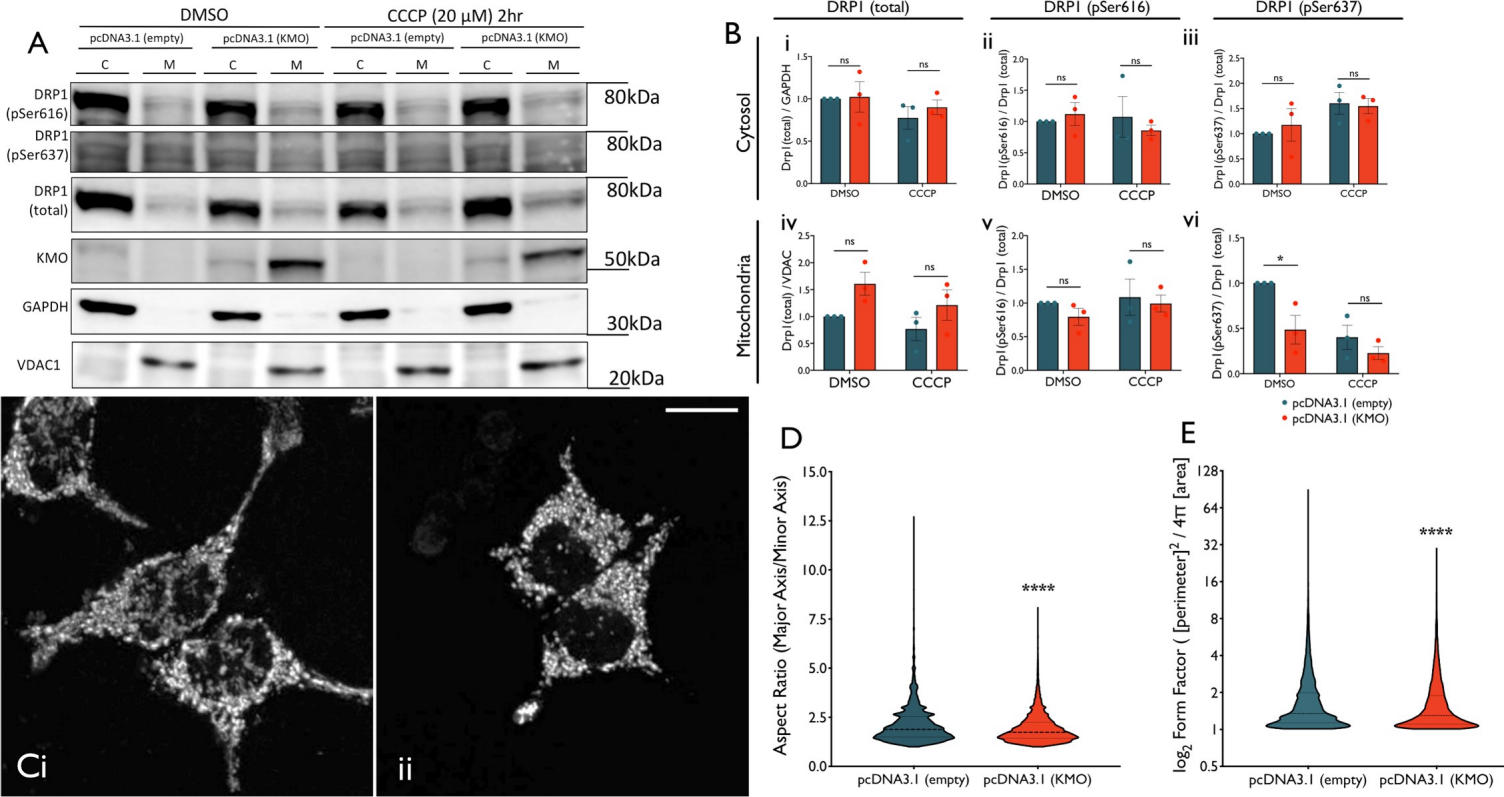
Mitochondrial DRP1 Ser637 phosphorylation is reduced by KMO overexpression, resulting in an increase in mitochondrial fission. **(A)** Immunoblotting against DRP1 (total, pSer616 and pSer637), KMO, GAPDH and VDAC1 in cytosolic and mitochondrial fractions of HEK 293T cell transfected with pcDNA3.1 (empty of KMO), treated with DMSO or CCCP (20 μM) for 2 hrs. **(B)** Total cytosolic DRP1 normalised to GAPDH levels by densitometry of immunoblot bands **(i)**. Cytosolic DRP1 pSer616 **(ii)** and pSer637 **(iii)** normalised to total cytosolic DRP1. Total mitochondrial DRP1 normalised to VDAC1 **(iv)**. Mitochondrial DRP1 pSer616 **(v)** and pSer637 **(vi)** normalised to total mitochondrial DRP1 (mean ± SD, two-way ANOVA, Tukey *post hoc*, *** *P* < 0.001, n = 3). **(C)** MitoTracker Red FM staining in HEK 293T cells transfected with pcDNA3.1 vector [empty (i) or KMO (ii)] (scale bar = 10 μm). **(D)** Aspect ratio of mitochondria from HEK 293T cells transfected with control pcDNA3.1 or pcDNA3.1 (KMO) vectors (Mann Whitney U Test, **** *P* < 0.0001). **(E)** Form Factor of mitochondria from HEK 293T cells transfected with control pcDNA3.1 or pcDNA3.1 (KMO) vectors (Mann Whitney U Test, **** *P* < 0.0001).

To investigate if KMO modulates DRP1 post-translational regulation, the phospho-status of these two residues was investigated in cytosolic and mitochondrial DRP1 in HEK 293T cells overexpressing hKMO (Fig 7A). Cells were treated with vehicle (DMSO) or CCCP (20 μM), which has been previously shown to decrease Ser637 phosphorylation of mitochondrial DRP1 [55]. DRP1 pSer637 in the mitochondrial fraction was significantly decreased in KMO-overexpressing cells treated with DMSO (Fig 7A and 7B). These findings suggest that KMO plays a role in the dephosphorylation of DRP1 at Ser637 under basal conditions, thereby promoting mitochondrial fission. To confirm this, we overexpressed KMO in HEK 293T cells and assessed mitochondrial morphology by Mitotracker Red staining (Fig 7C). Mitochondria had a smaller aspect ratio and form factor in KMO overexpressing cells compared to controls (Fig 7D and 7E), indicating that KMO overexpression induces DRP1-regulated mitochondrial fission.

## Discussion

KMO is a promising therapeutic target for the treatment of a number of human diseases, particularly those associated with chronic inflammation such as neurodegenerative disorders [1,2,56]. Given the emerging prominence of aberrant mitochondrial quality control in the pathology of several disorders including Huntington’s [57,58], Alzheimer’s [59] and Parkinson’s diseases [60–63], we felt it was important to investigate potential mitochondrial functions of KMO, which is localised to the OMM [64]. In an RNAi screen in *Drosophila* S2R+ cells, knockdown of the KMO-encoding gene *cn* resulted in elongated mitochondria [9]. This observation was replicated in this study, both in S2 cells and in *cn*-null flies *in vivo*. These flies also exhibit an increase in total mitochondrial mass, which is reflected by both an increase in total mitochondrial area coverage in the *Drosophila* retina and an increase in CS activity from whole-fly homogenates.

Despite an increase in mitochondrial mass, *cn*-null and RNAi knockdown flies have a decreased respiratory capacity of ETS complex I, suggesting impaired quality of this organelle. Citrate synthase activity and respiratory capacity are unaffected by supplementing flies with 3-HK at a concentration known to restore it to physiological levels suggesting that mitochondrial phenotypes are independent of KMO-mediated production of 3-HK. Notably, lifespan was reduced in *cn*^*3*^ flies compared to the Canton S wild-type strain, similar to a moderate decrease in lifespan previously reported in *cn*^*1*^ null mutants [65]. In contrast, *v*^*36f*^ mutants, which lack 3-HK production but also exhibit decreased KYNA [4,5], showed a significant increase in lifespan, an observation which has also been made upon knockdown of the *C*. *elegans* orthologue *tdo-2* [66]. The difference in phenotype observed upon *v* or *cn* LOF indicates that it is independent of 3-HK levels. Indeed, in the worm, *tdo-2* knockdown was found to convey enhanced lifespan through elevated TRP levels [66] and genetic / pharmacological inhibition of TRP metabolism via *v* or the ABC transporter *white* also enhances lifespan in *Drosophila* [67,68].

In our study, a decrease in startle induced locomotor activity was observed in *cn* knockdown and mutant flies. This phenotype has also been previously reported in adult *cn*^*1*^ amorphs compared to the Oregon R wild-type strain [65] and in *cn*^*1*^ larvae compared to the Canton S wild-type strain [69]. *v* LOF mutant larvae were also assayed and showed a locomotor phenotype, which was thus attributed to aberrant KP metabolite levels [69]. The phenotype was more apparent in *cn* than *v* mutants, which was suggested to be due to the greater accumulation of KYNA and subsequent inhibition of nicotinic acetylcholine receptors in *cn* mutants, although this was not tested. There is some evidence that elevated KYNA affects the viability of wild-type *Drosophila*, although this effect is dependent on 3-HK synthesis, thus not observed in *v* or *cn* amorphs and augmented in *cardinal* mutants [70]. The effect of elevated KYNA via KMO inhibition [3,71] on mitochondrial form and function nevertheless warrants further investigation.

The RNAi screen that identified *cn* as a modulator of mitochondrial morphology found that *cn* knockdown impaired the recruitment of dParkin-GFP to mitochondria in *Drosophila* S2R+ cells treated with the protonophore CCCP or the oxidative stress inducing pesticide paraquat [9]. The increase in mitochondrial mass, accompanied by a decrease in respiratory capacity in *cn* deficient flies, supports the hypothesis that KMO could be involved in mitochondrial quality control and turnover, which is governed by PINK1/PRKN-mediated mitophagy. Homozygous *cn* LOF causes partial lethality in *Pink1* and *parkin* mutant flies and modulates the penetrance of the defective thorax phenotype, suggesting some functional overlap between KMO and the mitophagy pathway. Overexpression of *cn* or *hKMO* robustly rescued climbing defects in *Pink1* mutant flies, a phenotype which has previously been rescued by overexpression of *Drp1* [72] and *parkin* [26,28,73]. Quantification of mitophagy using the fluorescent reporter systems mt-Keima [74] and mito-QC [75] revealed that mitophagy defects in *Pink1* and *parkin* mutant flies are not apparent in young adults [76,77] but are apparent by Day 30 [77]. It would be interesting to assess age-related changes in mitophagy in KMO-overexpressing *Pink1* and *parkin* mutants, to further investigate if improvements in mitophagy facilitate the rescue of climbing defects in *Pink1* mutants and lifespan in *Pink1* and *parkin* mutants. Overexpression of *cn* had no effect on the levels of Marf however, indicating that any effect is independent of Parkin recruitment, ubiquitination and proteasomal degradation of its targets.

Heterozygous *Drp1* LOF or a dominant negative *Drp1* allele both cause lethality in *Pink1* and *parkin* mutants [31]. This is thought to be due to DRP1-mediated fission facilitating mitophagy, by producing smaller mitochondria that are easily engulfed by autophagosomes[34,78]. This could mean that alternative routes to mitophagy, such as via the mitochondrial ubiquitin ligase MUL1 [79], are sufficient in the absence of PINK1 or Parkin in a *Drp1*^*+/+*^, but not a *Drp1*^*+/-*^ background. Given the elongated mitochondria observed in KMO deficient *Drosophila* in this study and the interplay observed between KMO and DRP1, the partial lethality of *cn* LOF in *Pink1* and *parkin* mutants is likely to be caused through a DRP1-dependant mechanism.

The mechanism by which KMO is modulating mitochondrial DRP1 Ser637 phosphorylation should also be investigated further. Given the lack of kinase or phosphatase-like domains present in KMO, direct modulation of phosphorylation status can be excluded as a possibility. A simple, but perhaps improbable explanation, is that KMO interacts directly with DRP1 in a manner similar to mitochondrial fission factor (MFF), FIS1, mitochondrial dynamic proteins of 49 and 51 kDa (MiD49 and MiD51) [55]. This interaction might be influenced by DRP1 phosphorylation status or might promote the dephosphorylation of DRP1 Ser637. Interactions between DRP1 and its mitochondrial recruiters (MFF, FIS1, MiD49 and MiD5) could also be investigated in a KMO overexpression context in HEK 293T cells, as KMO could cause a (de)stabilization of one or several of these interactions. Indeed, an increase in mitochondrial DRP1 and DRP1 pSer637 was observed in HeLa cells overexpressing MiD49 or MiD51 [55]. These cells exhibited elongated mitochondria but underwent rapid fission upon CCCP treatment. This was interpreted by the authors as the selective recruitment of inactive DRP1 pSer637 to mitochondria by MiD49/51, thus priming organelles for more efficient fission upon mitochondrial damage. This is further supported by the observation that MiD49/51 double knockout cells are resistant to CCCP induced mitochondrial fission [80]. Conversely, MFF cannot bind DRP1 pSer637 and overexpression of MFF leads to increased mitochondrial fission under basal conditions, indicating that MFF recruits the active form of DRP1 [81]. The interaction between DRP1 and MFF is enhanced upon UV-irradiation induced apoptosis and is accompanied by a decrease in DRP1 pSer637, a decrease in DRP1-MiD51 interactions and an increase in FIS1-MiD51 interactions [81]. This gives an insight into the complex interactions between DRP1 mitochondrial recruiters, in the priming and triggering of mitochondrial fission. Given the decrease in mitochondrial DRP1 pSer637 observed upon KMO overexpression, the interactions between MiD51 and DRP1/FIS1 could give further insight into the mechanisms by which KMO regulates DRP1.

Intriguingly and counterintuitively, KMO overexpression in HEK 293T cells has been shown to be protective against 3-HK mediated loss of mitochondrial membrane potential [82]. This protection was abolished upon inhibition of KMO enzymatic activity, or knockdown of downstream enzymes kynureninase (KYNU) and quinolinic acid phosphoribosyltransferease (QPRT), both of which are upregulated upon KMO overexpression, revealing complex feedback mechanisms operating in the KP. Therefore, the effect(s) of KMO overexpression on mitochondrial DRP1 could be further explored by pharmacological KMO inhibition or knockdown of KYNU/QPRT, which would clarify whether these observations are a direct effect of KMO protein or due to feedback mechanisms operating within the KP. Again, elevated KYNA cannot be excluded as the cause of this effect, potentially via alterations in Ca^2+^ signalling due to GPR35 activation [83], resulting in activation of calcineurin and dephosphorylation of DRP1 [54].

**Fig 8:**
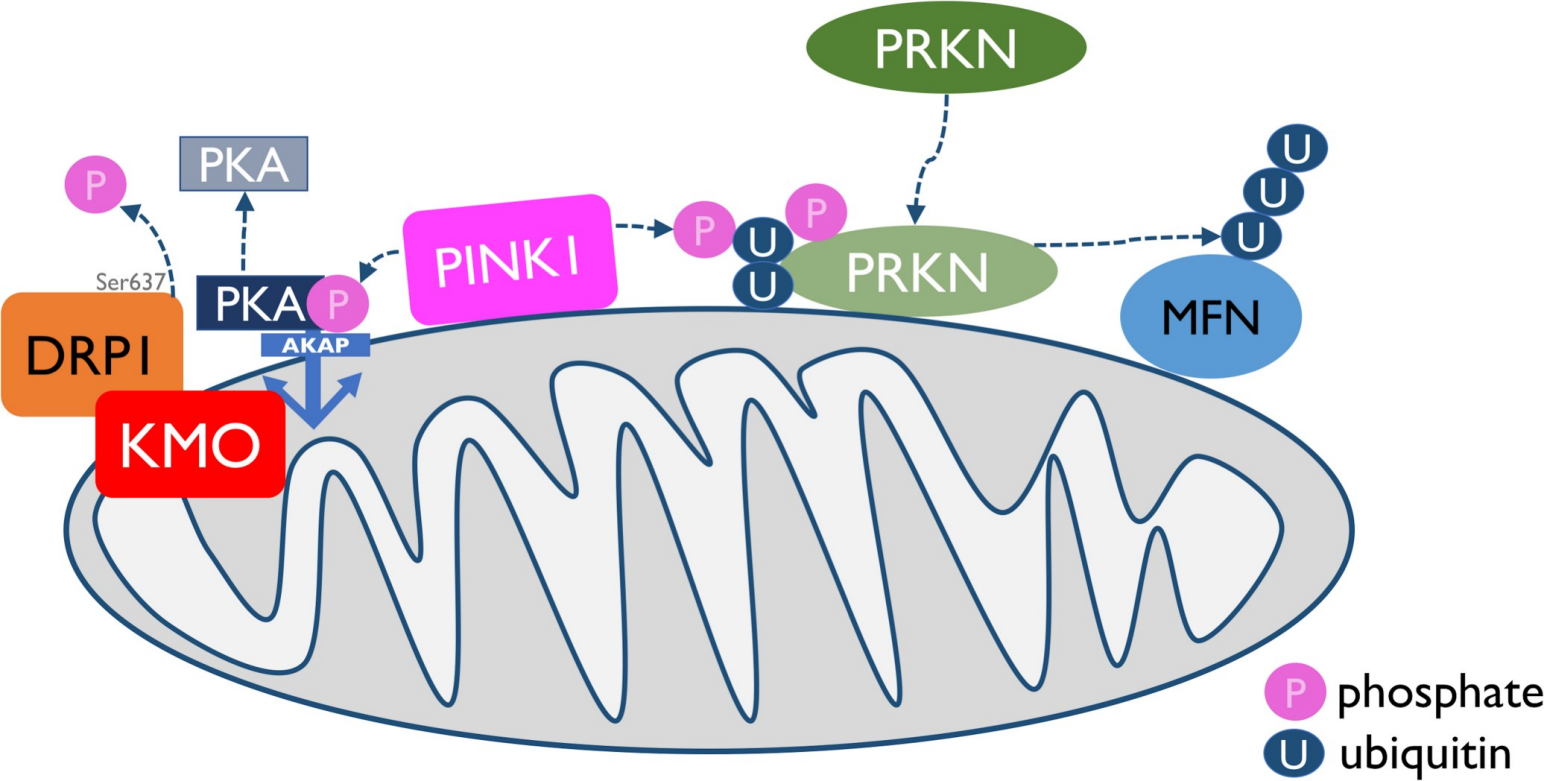
KMO regulates mitochondrial dynamics by modulating DRP1 phosphorylation. PINK1/PRKN mediated mitophagy is tightly linked to mitochondrial dynamics, via PINK1 phosphorylation of AKAP, leading to dephosphorylation of Ser637 of DRP1. Overexpression of KMO has a similar effect on DRP1 phosphorylation levels, indicating a mechanism for KMO interaction with both mitochondrial dynamics and mitophagy mechanisms.

Taken together, this study suggests a novel role of KMO in mitochondrial form and function. Functional interactions with PINK1, PRKN and DRP1 implicate KMO in mechanisms associated with mitochondrial dysfunction in neurodegeneration, such as defects in mitochondrial morphology and mitophagy (Fig 8). Future work will be required to fully tease apart the mechanistic underpinnings of these novel observations.

## Supporting information

S1 Fig (A) *cinnabar* and *parkin* mRNA levels are reduced in S2 cells upon dsRNA knockdown. Values represent normalised mRNA levels of target gene (*cinnabar* or *parkin*) in dsRNA treated cells compared to *f.luc* dsRNA treated controls (mean ± SD; pairwise fixed reallocation randomization test, *** *P* < 0.001, n = 3). (B) *cinnabar* mRNA levels are reduced in Drosophila upon RNAi knockdown. *cn* mRNA level relative to the reference gene *rp49*, normalised to that of the RNAi control (mean ± SD, pairwise fixed reallocation randomization test. Ten flies per n, n = 3). (C) OXPHPOS is reduced upon cn knockdown. Respiratory capacity is reduced in *cn^RNAi^* flies (mean ± SEM; paired t test, Holm-Sidak post hoc, * *P* < 0.05, n = 7). (D) Climbing ability is reduced upon *cn* knockdown. *cn^RNAi^; GAL4^Act5C^* compared to the RNAi control group. Ability was assessed using the rapid iterative negative geotaxis (RING) assay. 10 flies were placed inside a 20 cm vial and tapped to the bottom. The percentage of flies that passed a 8 cm threshold line after 10 s was counted (mean ± SEM; two-way ANOVA, Sidak *post hoc*. 10 flies per n, n = 5–10).
(TIF)
