# Supplementary figures and images for: A novel role for kynurenine 3-monooxygenase in mitochondrial dynamics
Source: PLoS Genet. 2020 Nov 10;16(11):e1009129. doi: 10.1371/journal.pgen.1009129 (PMC7654755; doi:10.1371/journal.pgen.1009129)

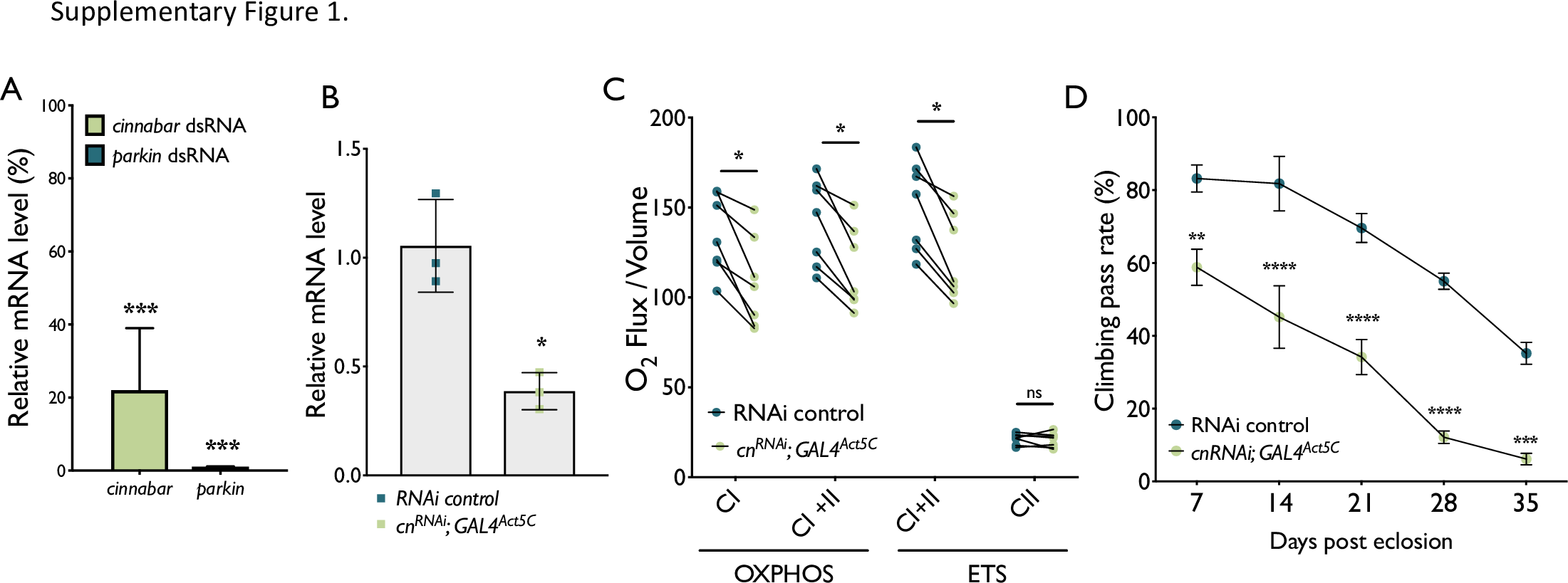

Supplement: S1 Fig — (A) cinnabar and parkin mRNA levels are reduced in S2 cells upon dsRNA knockdown. Values represent normalised mRNA levels of target gene (cinnabar or parkin) in dsRNA treated cells compared to f.luc dsRNA treated controls (mean ± SD; pairwise fixed reallocation randomization test, *** P < 0.001, n = 3). (B) cinnabar mRNA levels are reduced in Drosophila upon RNAi knockdown. cn mRNA level relative to the reference gene rp49, normalised to that of the RNAi control (mean ± SD, pairwise fixed reallocation randomization test. Ten flies per n, n = 3). (C) OXPHPOS is reduced upon cn knockdown. Respiratory capacity is reduced in cnRNAi flies (mean ± SEM; paired t test, Holm-Sidak post hoc, * P < 0.05, n = 7). (D) Climbing ability is reduced upon cn knockdown. cnRNAi; GAL4Act5C compared to the RNAi control group. Ability was assessed using the rapid iterative negative geotaxis (RING) assay. 10 flies were placed inside a 20 cm vial and tapped to the bottom. The percentage of flies that passed a 8 cm threshold line after 10 s was counted (mean ± SEM; two-way ANOVA, Sidak post hoc. 10 flies per n, n = 5–10). (TIF) [file pgen.1009129.s001.tif]
